# Supplementary material for: Genome-Wide Association Analysis and Genetic Parameters for Feed Efficiency and Related Traits in Yorkshire and Duroc Pigs
Source: Animals (Basel). 2022 Jul 26;12(15):1902. doi: 10.3390/ani12151902 (PMC9329986; doi:10.3390/ani12151902)
Supplement: Supplementary file 1 [file animals-12-01902-s001.zip › animals-1762235-supplementary.pdf]

Table S1 Number of observations (N), means (standard deviations) of deregressed estimated breeding values (DEBV)

Traits[1]	Yorkshire		Duroc	
	N	Means ± SD	N	Means ± SD
ADFI	880	0.086±0.360	485	0.021±0.372
ADG	880	0.006±0.100	485	-0.001±0.081
FCR	880	0.022±0.301	485	-0.013±0.242
RFI	880	-0.024±0.577	485	-0.033±0.185

[1]FCR: Feed conversion ratio, ADG: Average daily gain, ADFI: Average daily feed intake, RFI: Residual feed intake.

Table S2. Identification of genome-wide or suggestive significant SNPs that associated with FE and related traits in pigs

Breed[1]	Traits[2]	Chr.	SNP ID	Location(bp)[3]	Allele[4]	MAF[5]	P-value[6]	pigQTLdb[7]
DD	ADFI	18	seq-rs327709242	32623286	C/T	0.282	2.52E-05	-
DD	ADG	3	seq-rs342109728	79049546	T/C	0.419	3.87E-06	ADG
DD	ADG	11	seq-rs321691240	67792739	A/G	0.419	3.08E-06	-
DD	ADG	13	seq-rs710999761	80501143	T/G	0.469	2.27E-07	ADG
DD	ADG	13	seq-rs334871208	98302557	T/C	0.296	8.75E-07	ADG
DD	ADG	14	seq-rs80921027	64144092	A/G	0.282	1.77E-06	ADFI
DD	ADG	15	seq-rs80957899	86124988	G/T	0.495	2.69E-05	ADG
DD	ADG	15	seq-rs328663154	101376032	C/A	0.13	5.99E-06	ADG
DD	FCR	3	seq-rs342109728	79049546	T/C	0.419	2.67E-05	ADG
DD	FCR	15	seq-rs329844461	16281234	C/T	0.419	7.36E-07	-
DD	RFI	8	seq-rs345393699	89446476	G/A	0.428	3.26E-05	-
YY	ADFI	1	seq-rs80843328	160210902	G/A	0.411	2.05E-05	ADG
YY	ADFI	1	seq-rs320681260	160883673	G/A	0.411	1.31E-05	ADG
YY	ADFI	1	seq-rs80997722	161540913	A/G	0.41	1.52E-05	ADG
YY	ADFI	1	seq-rs330215372	161583241	A/G	0.41	1.52E-05	ADG
YY	ADFI	1	seq-rs337097929	162292957	G/A	0.403	2.51E-05	ADG
YY	ADFI	1	seq-rs80988025	162345362	G/A	0.403	2.51E-05	ADG
YY	ADFI	1	seq-rs333500003	162776275	C/T	0.398	2.59E-05	ADG
YY	ADFI	1	seq-rs330637696	162783329	T/C	0.399	1.82E-05	ADG
YY	ADFI	3	seq-rs334252973	83351277	C/T	0.291	1.47E-05	ADG
YY	ADFI	4	seq-rs322234522	69687124	T/C	0.048	6.32E-06	FCR、ADG
YY	ADFI	6	seq-rs320347867	105104215	A/G	0.048	1.11E-05	ADFI、ADG
YY	ADFI	12	seq-rs81344522	4675208	T/C	0.096	2.98E-05	ADG
YY	ADFI	13	seq-rs705817794	38267479	A/G	0.047	1.79E-05	ADG
YY	ADFI	13	seq-rs793013452	44606060	C/A	0.047	2.03E-05	ADG
YY	ADG	1	seq-rs344383954	115356348	C/A	0.046	1.32E-06	ADG
YY	ADG	3	seq-rs334252973	83351277	C/T	0.291	5.46E-07	ADG
YY	ADG	4	seq-rs80795362	27377224	G/A	0.062	1.51E-05	ADG
YY	ADG	4	seq-rs322234522	69687124	T/C	0.048	1.58E-09	FCR、ADG
YY	ADG	6	seq-rs81475738	80622284	G/T	0.167	5.74E-06	FCR、ADG
YY	ADG	6	seq-rs320347867	105104215	A/G	0.048	1.29E-09	ADFI、ADG

YY	ADG	8	seq-rs339132738	53757344	T/C	0.052	2.92E-08	ADG
YY	ADG	13	seq-rs705817794	38267479	A/G	0.047	1.6E-09	ADG
YY	ADG	13	seq-rs793013452	44606060	C/A	0.047	2.03E-09	ADG
YY	ADG	13	seq-rs705621029	147609391	A/C	0.051	6.61E-08	FCR、ADG
YY	ADG	13	seq-rs338850979	158150159	T/C	0.047	2.56E-08	FCR、ADG
YY	ADG	14	seq-rs80790167	66511894	T/G	0.051	9.32E-08	ADFI
YY	ADG	15	seq-rs699198332	57776636	A/G	0.058	8.52E-08	FCR
YY	RFI	2	seq-rs320243411	66008692	C/A	0.271	2.51E-06	ADG
YY	RFI	2	seq-rs81272049	66370420	G/A	0.305	2.89E-05	ADG
YY	RFI	2	seq-rs322933932	66729446	G/A	0.271	2.51E-06	ADG
YY	RFI	2	seq-rs330639556	66849146	C/A	0.272	1.75E-06	ADG
YY	RFI	2	seq-rs333725490	67397353	A/G	0.306	1.34E-05	ADG
YY	RFI	2	seq-rs343447412	69408667	C/A	0.258	2.58E-05	ADG
YY	RFI	2	seq-rs331867151	71484107	T/C	0.247	2.86E-06	ADG
YY	RFI	2	seq-rs336484525	71484462	G/C	0.247	2.86E-06	ADG
YY	RFI	2	seq-rs340791819	72523054	C/T	0.255	2.06E-05	ADG
YY	RFI	2	seq-rs81223451	72820932	T/C	0.253	2.97E-05	ADG
YY	RFI	2	seq-rs324255146	72934295	T/C	0.253	2.97E-05	ADG
YY	RFI	2	seq-rs345488861	73162496	C/T	0.253	2.97E-05	ADG
YY	RFI	2	seq-rs320237095	73217053	A/G	0.243	1.85E-06	ADG
YY	RFI	2	seq-rs712612698	73232214	C/T	0.253	2.97E-05	ADG
YY	RFI	2	seq-rs712037651	73264304	A/G	0.253	2.97E-05	ADG
YY	RFI	2	seq-rs792542846	73363418	T/C	0.243	1.85E-06	ADG
YY	RFI	2	seq-rs335984226	73428891	T/C	0.243	1.85E-06	ADG
YY	RFI	2	seq-rs81225998	73477969	T/C	0.253	2.97E-05	ADG
YY	RFI	2	seq-rs329767211	73697944	C/T	0.253	2.97E-05	ADG
YY	RFI	2	seq-rs693098203	73817678	C/T	0.243	1.85E-06	ADG
YY	RFI	2	seq-rs329056098	73818550	C/T	0.243	1.85E-06	ADG
YY	RFI	2	seq-rs344662679	76061262	T/C	0.242	1.73E-05	ADG
YY	RFI	15	seq-rs338952192	119660194	C/T	0.476	2.84E-05	ADG

[1]YY, Yorkshire; DD, Duroc.

[2]ADFI, average daily feed intake; ADG, average daily gain; FCR, feed conversion rate; RFI, residual feed intake.

[3]physical SNP position (Sus scrofa 11.1 genome version)

[4]major/minor allele.

[5]Minor Allele Frequency.

[6]*P*-value of Wald test for additive variance of FE traits.

[7]Whether these SNPs located in the QTL region of some traits of pigQTLdb (https://www.animalgenome.org/cgi-bin/QTLdb/SS/index)

Table S3. Identification of QTL regions that associated with FE and related traits in pigs

Breed[1]	Trait[2]	Region (bp)[3]	nSNP[4]	SNP list[5]
YY	RFI	2:72023054-74318550	13	seq-rs340791819,seq-rs81223451, seq-rs324255146,seq-rs345488861, seq-rs320237095,seq-rs712612698, seq-rs712037651,seq-rs792542846, seq-rs335984226,seq-rs81225998, seq-rs329767211,seq-rs693098203, seq-rs329056098
YY	ADFI	1:159710902-163283329	8	seq-rs80843328,seq-rs320681260, seq-rs80997722,seq-rs330215372, seq-rs337097929,seq-rs80988025, seq-rs333500003,seq-rs330637696
YY	RFI	2:65508692-67897353	5	seq-rs320243411,seq-rs81272049, seq-rs322933932,seq-rs330639556, seq-rs333725490
YY	RFI	2:70984107-71984462	2	seq-rs331867151,seq-rs336484525
YY	ADG	1:114856348-115856348	1	seq-rs344383954
YY	ADG	3:82851277-83851277	1	seq-rs334252973
YY	ADG	4:26877224-27877224	1	seq-rs80795362
YY	ADG	4:69187124-70187124	1	seq-rs322234522
YY	ADG	6:80122284-81122284	1	seq-rs81475738
YY	ADG	6:104604215-105604215	1	seq-rs320347867
YY	ADG	8:53257344-54257344	1	seq-rs339132738
YY	ADG	13:37767479-38767479	1	seq-rs705817794
YY	ADG	13:44106060-45106060	1	seq-rs793013452
YY	ADG	13:147109391-148109391	1	seq-rs705621029
YY	ADG	13:157650159-158650159	1	seq-rs338850979
YY	ADG	14:66011894-67011894	1	seq-rs80790167
YY	ADG	15:57276636-58276636	1	seq-rs699198332
YY	ADFI	3:82851277-83851277	1	seq-rs334252973
YY	ADFI	4:69187124-70187124	1	seq-rs322234522
YY	ADFI	6:104604215-105604215	1	seq-rs320347867
YY	ADFI	12:4175208-5175208	1	seq-rs81344522
YY	ADFI	13:37767479-38767479	1	seq-rs705817794
YY	ADFI	13:44106060-45106060	1	seq-rs793013452
YY	RFI	2:68908667-69908667	1	seq-rs343447412
YY	RFI	2:75561262-76561262	1	seq-rs344662679
YY	RFI	15:119160194-120160194	1	seq-rs338952192
DD	FCR	3:78549546-79549546	1	seq-rs342109728
DD	FCR	15:15781234-16781234	1	seq-rs329844461
DD	ADG	3:78549546-79549546	1	seq-rs342109728
DD	ADG	11:67292739-68292739	1	seq-rs321691240
DD	ADG	13:80001143-81001143	1	seq-rs710999761
DD	ADG	13:97802557-98802557	1	seq-rs334871208
DD	ADG	14:63644092-64644092	1	seq-rs80921027
DD	ADG	15:85624988-86624988	1	seq-rs80957899
DD	ADG	15:100876032-101876032	1	seq-rs328663154

DD	ADFI	18:32123286-33123286	1	seq-rs327709242
DD	RFI	8:88946476-89946476	1	seq-rs345393699

[1]YY, Yorkshire; DD, Duroc.

[2]ADFI, average daily feed intake; ADG, average daily gain; FCR, feed conversion rate; RFI, residual feed intake.

[3]chromosome:start:end (Sus scrofa 11.1 genome version).

[4]Number of SNPs located in QTL region.

[5]ID of SNPs located in QTL region.

Table S4. Candidate genes that associated with FE and related traits in pigs

Breed[1]	Traits[2]	Gene name	Chr.	Gene start (bp)[3]	Gene end (bp)	Reference[4]
YY	ADFI	ATP8B1	1	162933131	163063032	[1]
YY	ADFI	LMAN1	1	161607139	161631650	[2]
YY	RFI	CARM1	2	69602130	69644862	
YY	ADFI	MC4R	1	160772013	160774124	[3-6]
YY	ADG	CYP7B1	4	69616035	69806496	
YY	ADFI	CYP7B1	4	69616035	69806496	
YY	RFI	DENND1C	2	72610566	72620212	
YY	RFI	DIRAS1	2	75908509	75929152	
YY	ADG	EPHA8	6	80516428	80550588	
YY	RFI	FARSA	2	66105428	66118191	
YY	RFI	FBXW9	2	66291172	66297215	
YY	RFI	AP3D1	2	76428436	76471258	[7]
YY	RFI	DNM2	2	69474031	69574424	[8]
YY	RFI	INSR	2	71797204	71936139	[9-11]
YY	RFI	MRPL4	2	69063626	69082692	
YY	RFI	LONP1	2	73266258	73286774	[12]
YY	RFI	MYDGF	2	74185037	74202301	
YY	RFI	NDUFA11	2	73130403	73137916	
YY	RFI	OR7D2	2	67372003	67375663	
YY	RFI	PLIN4	2	74316771	74338941	[13]
YY	RFI	SLC44A2	2	69364989	69399081	
YY	ADG	SLC9C1	13	147517663	147603247	
YY	ADFI	ST6GALNAC1	12	4890406	4909351	
YY	RFI	STX10	2	65914057	65917073	
YY	RFI	SYCE2	2	66128492	66131447	
YY	RFI	TIMM44	2	71271796	71290546	[14]
YY	ADG	TOMM70	13	158577377	158628267	
DD	ADFI	BMT2	18	32798289	32900767	[15]
DD	ADFI	GPR85	18	32667486	32672746	[15]
DD	ADFI	TMEM168	18	32927754	32957985	[15]
DD	ADG	FARP1	11	67144103	67457548	[16]
DD	ADG	UBAC2	11	68145101	68319939	[17]
DD	FCR	MCM6	15	15917042	15985405	[18]
DD	FCR	ZRANB3	15	16414757	16727842	[19-21]
DD	RFI	PCDH18	8	89321491	89335395	[22]

[1]YY, Yorkshire; DD, Duroc.

[2]ADFI, average daily feed intake; ADG, average daily gain; FCR, feed conversion rate; RFI, residual feed intake.

[3]physical position (Sus scrofa 11.1 genome version)

[4]Articles that reported association between the genes and traits of livestock.

Table S5. Top 30 terms or pathways ranked by corrected *P*-Value in enrichment analysis

Breed[1]	Traits[2]	Database[3]	Term	Corrected *P*-Value
YY	ADG	GO	neuron remodeling	0.005602
YY	ADFI	GO	sialylation	0.00837
YY	ADFI	GO	protein monoubiquitination	0.011214
DD	FCR	GO	hydrolase activity, hydrolyzing O-glycosyl compounds	0.011813
DD	FCR	GO	MKS complex	0.011813
DD	FCR	GO	aminoacyl-tRNA synthetase multienzyme complex	0.011813
DD	FCR	GO	ciliary transition zone	0.011813
DD	FCR	GO	ciliary membrane	0.011813
DD	FCR	KEGG	Galactose metabolism	0.01567
DD	FCR	KEGG	Carbohydrate digestion and absorption	0.01567
DD	FCR	GO	non-motile cilium assembly	0.01567
DD	FCR	GO	smoothened signaling pathway	0.016788
DD	FCR	KEGG	Aminoacyl-tRNA biosynthesis	0.018415
YY	ADFI	GO	nuclear body	0.019464
DD	FCR	GO	carbohydrate metabolic process	0.028723
DD	ADG	GO	mitochondrial matrix	0.035277
DD	ADG	GO	germinal vesicle	0.035277
DD	ADG	GO	meiotic spindle organization	0.035277
DD	ADG	GO	positive regulation of meiotic cell cycle process involved in oocyte maturation	0.035277
DD	ADG	GO	positive regulation of synaptic transmission	0.035277
DD	ADG	GO	protein localization to kinetochore	0.035277
DD	ADG	GO	histone phosphorylation	0.035277
DD	ADG	GO	ubiquinone binding	0.035277
DD	ADG	GO	cellular respiration	0.035277
DD	ADG	GO	MKS complex	0.035277
DD	ADG	GO	RNA polymerase II CTD heptapeptide repeat kinase activity	0.035277
DD	ADG	GO	Rac guanyl-nucleotide exchange factor activity	0.035277
DD	ADG	GO	ciliary transition zone	0.035277
DD	ADG	GO	positive regulation of protein localization to nucleus	0.035277
DD	ADG	GO	translation elongation factor activity	0.035277

[1]YY, Yorkshire; DD, Duroc.

[2]ADFI, average daily feed intake; ADG, average daily gain; FCR, feed conversion rate; RFI, residual feed intake.

[3]GO, Gene Ontology; KEGG, Kyoto Encyclopedia of Genes and Genomes Pathway

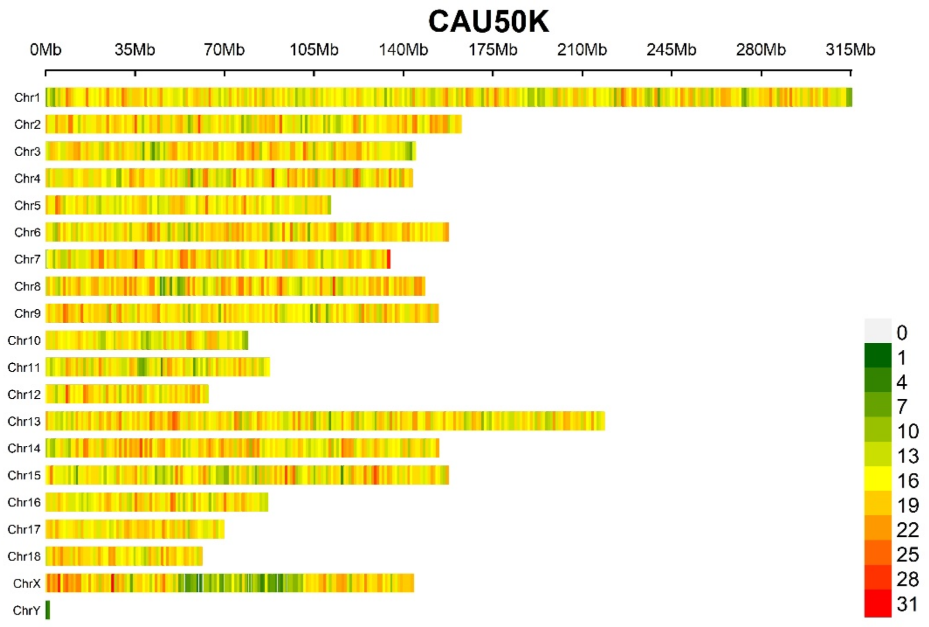

Figure S1. SNP-density plot of CAU50K SNP chip array; colors represent number of SNP within 1 Mbp

References:

1. Gozalo-Marcilla, M.; Buntjer, J.; Johnsson, M.; Batista, L.; Diez, F.; Werner, C.R.; Chen, C.; Gorjanc, G.; Mellanby, R.J.; Hickey, J.M., et al. Genetic architecture and major genes for backfat thickness in pig lines of diverse genetic backgrounds. *Genet Sel Evol* **2021**, 53, 76, doi:10.1186/s12711-021-00671-w.
2. Borowska, A.; Reyer, H.; Wimmers, K.; Varley, P.F.; Szwaczkowski, T. Detection of pig genome regions determining production traits using an information theory approach. *Livest Sci* **2017**, 205, 31-35, doi:https://doi.org/10.1016/j.livsci.2017.09.012.
3. Davoli, R.; Braglia, S.; Valastro, V.; Annaratone, C.; Comella, M.; Zambonelli, P.; Nisi, I.; Gallo, M.; Buttazzoni, L.; Russo, V. Analysis of MC4R polymorphism in Italian Large White and Italian Duroc pigs: association with carcass traits. *Meat Sci* **2012**, 90, 887-892, doi:10.1016/j.meatsci.2011.11.025.
4. Kim, K.S.; Larsen, N.; Short, T.; Plastow, G.; Rothschild, M.F. A missense variant of the porcine melanocortin-4 receptor (MC4R) gene is associated with fatness, growth, and feed intake traits. *Mamm Genome* **2000**, 11, 131-135.
5. Silva, É.F.; Lopes, M.S.; Lopes, P.S.; Gasparino, E. A genome-wide association study for feed efficiency-related traits in a crossbred pig population. *Animal* **2019**, 13, 2447-2456, doi:10.1017/S1751731119000910.
6. Thuy, H.T.; Nhan, G.T.T.; Mai, P.T.P.; Thuy, T.T.T.; Nam, L.Q.; Thuy, D.P.; Van Hung, N.; Manh, T.X.; Van Soan, D.; Lan, P.D. Associations of some candidate gene polymorphisms with growth traits in Duroc pigs. *Livestock Research for Rural Development* **2019**, 31.
7. Ding, R.; Zhuang, Z.; Qiu, Y.; Ruan, D.; Wu, J.; Ye, J.; Cao, L.; Zhou, S.; Zheng, E.; Huang, W.,

et al. Identify known and novel candidate genes associated with backfat thickness in Duroc pigs by large-scale genome-wide association analysis. *J Anim Sci* **2022**, 100, c12, doi:10.1093/jas/skac012.
8. Kong, R.S.G.; Liang, G.; Chen, Y.; Stothard, P.; Guan, L.L. Transcriptome profiling of the rumen epithelium of beef cattle differing in residual feed intake. *Bmc Genomics* **2016**, 17, 592, doi:10.1186/s12864-016-2935-4.
9. Duy, N.D.; Ostersen, T.; Strathe, A.B.; Mark, T.; Jensen, J.; Kadarmideen, H.N. Genome-wide association and systems genetic analyses of residual feed intake, daily feed consumption, backfat and weight gain in pigs. *Bmc Genet* **2014**, 15, doi:10.1186/1471-2156-15-27.
10. Gondret, F.; Vincent, A.; Houée-Bigot, M.; Siegel, A.; Lagarrigue, S.; Causeur, D.; Gilbert, H.; Louveau, I. A transcriptome multi-tissue analysis identifies biological pathways and genes associated with variations in feed efficiency of growing pigs. *Bmc Genomics* **2017**, 18, 244, doi:10.1186/s12864-017-3639-0.
11. Yu, J.; Zhao, P.; Zheng, X.; Zhou, L.; Wang, C.; Liu, J. Genome-Wide Detection of Selection Signatures in Duroc Revealed Candidate Genes Relating to Growth and Meat Quality. *G3 Genes|Genomes|Genetics* **2020**, 10, 3765-3773, doi:10.1534/g3.120.401628.
12. Moreira, G.C.M.; Poleti, M.D.; Pértille, F.; Boschiero, C.; Cesar, A.S.M.; Godoy, T.F.; Ledur, M.C.; Reecy, J.M.; Garrick, D.J.; Coutinho, L.L. Unraveling genomic associations with feed efficiency and body weight traits in chickens through an integrative approach. *Bmc Genet* **2019**, 20, 83, doi:10.1186/s12863-019-0783-3.
13. Zappaterra, M.; Mazzoni, M.; Zambonelli, P.; Davoli, R. Investigation of the Perilipin 5 gene expression and association study of its sequence polymorphism with meat and carcass quality traits in different pig breeds. *Animal* **2018**, 12, 1135-1143.
14. Mohammadi, H.; Rafat, S.A.; Moradi Shahrbabak, H.; Shodja, J.; Moradi, M.H. Genome-wide association study and gene ontology for growth and wool characteristics in Zandi sheep. *Journal of Livestock Science and Technologies* **2020**, 8, 45-55.
15. Jahuey-Martinez, F.J.; Parra-Bracamonte, G.M.; Sifuentes-Rincon, A.M.; Moreno-Medina, V.R. Signatures of selection in Charolais beef cattle identified by genome-wide analysis. *J Anim Breed Genet* **2019**, 136, 378-389, doi:10.1111/jbg.12399.
16. Gheyas, A.A.; Vallejo-Trujillo, A.; Kebede, A.; Lozano-Jaramillo, M.; Dessie, T.; Smith, J.; Hanotte, O. Integrated Environmental and Genomic Analysis Reveals the Drivers of Local Adaptation in African Indigenous Chickens. *Mol Biol Evol* **2021**, 38, 4268-4285, doi:10.1093/molbev/msab156.
17. Silva, D.B.S.; Fonseca, L.F.S.; Pinheiro, D.G.; Magalhães, A.F.B.; Muniz, M.M.M.; Ferro, J.A.; Baldi, F.; Chardulo, L.A.L.; Schnabel, R.D.; Taylor, J.F., et al. Spliced genes in muscle from Nelore Cattle and their association with carcass and meat quality. *Sci Rep-Uk* **2020**, 10, 14701, doi:10.1038/s41598-020-71783-4.
18. Yang, Y.; Zhou, H.; Hou, L.; Xing, K.; Shu, H. Transcriptional profiling of skeletal muscle reveals starvation response and compensatory growth in Spinibarbus hollandi. *Bmc Genomics* **2019**, 20, 938, doi:10.1186/s12864-019-6345-2.
19. Barendse, W.; Harrison, B.E.; Bunch, R.J.; Thomas, M.B.; Turner, L.B. Genome wide signatures of positive selection: the comparison of independent samples and the identification of regions associated to traits. *Bmc Genomics* **2009**, 10, 178, doi:10.1186/1471-2164-10-178.
20. Gibbs, R.A.; Taylor, J.F.; Van Tassell, C.P.; Barendse, W.; Eversole, K.A.; Gill, C.A.; Green, R.D.; Hamernik, D.L.; Kappes, S.M.; Lien, S., et al. Genome-wide survey of SNP variation uncovers the

genetic structure of cattle breeds. *Science* **2009**, 324, 528-532, doi:10.1126/science.1167936.
21. Gouveia, J.J.D.S.; Silva, M.V.G.B.; Paiva, S.R.; Oliveira, S.M.P.D. Identification of selection signatures in livestock species. *Genet Mol Biol* **2014**, 37, 330-342.
22. De Lima, A.O.; Koltes, J.E.; Diniz, W.J.; De Oliveira, P.S.; Cesar, A.S.; Tizioto, P.C.; Afonso, J.; de Souza, M.M.; Petrini, J.; Rocha, M.I. Potential biomarkers for feed efficiency-related traits in nelore cattle identified by co-expression network and integrative genomics analyses. *Front Genet* **2020**, 11, 189.
